# Lumboperitoneal shunt surgery *via* continuous two-stage procedure: Technique notes and outcomes

**DOI:** 10.3389/fneur.2022.1059316

**Published:** 2022-12-06

**Authors:** Zhao Li, Hao Wang, Han Zhang, Jiqi Yang, Xiaofeng Yang, Liang Wen

**Affiliations:** ^1^Shengzhou Hospital of Traditional Chinese Medicine, Shengzhou, China; ^2^First Affiliated Hospital, School of Medicine, Zhejiang University, Hangzhou, Zhejiang, China; ^3^Shengzhou People's Hospital, First Affiliated Hospital, School of Medicine, Zhejiang University, Shengzhou, China

**Keywords:** hydrocephalus, lumboperitoneal shunt, rehabilitation, shunt implantation, cerebrospinal fluid

## Abstract

**Background:**

Lumboperitoneal shunt (LPS) is an effective procedure for managing communicating hydrocephalus. LPS is performed as a one-stage procedure with the patient placed in the lateral position or as a two-stage procedure in which the patient's position is changed. Both methods can be bothersome to neurosurgeons. We designed a continuous two-stage LPS procedure in which the operative sites did not need to be sterilized again, and the surgical drapes did not have to be changed after changing the patient's position. In this study, we analyzed this procedure in terms of the technical features and outcomes.

**Methods:**

All patients from our institute who underwent LPS using the continuous two-stage procedure from October 2019 to August 2021 were reviewed retrospectively. The patient's demographic information, clinical features, operative data, and outcomes were analyzed.

**Results:**

A total of 46 consecutive patients who underwent LPS using the continuous two-stage procedure were enrolled. The mean operative duration was 70.6 ± 12.7 min. The 180-day revision rate for these patients was 2.2% (1/46). Moreover, 76.1% of the patients (35/46) experienced clinical improvement after LPS during the 180-day follow-up, and 70.0% of the patients (32/46) experienced an improvement in neuroimaging.

**Conclusion:**

We described a continuous two-stage LPS procedure. This method simplified the two-stage LPS procedure and maintained a low malfunction rate and shunt infection rate in our series.

## Introduction

Lumboperitoneal shunt (LPS) is an effective procedure for managing communicating hydrocephalus. LPS is preferred by patients compared with the ventriculoperitoneal shunt (VPS) because LPS does not require opening the cranium or puncturing the ventricle. Several clinical studies have confirmed that LPS is as effective as VPS in patients with hydrocephalus, including idiopathic normal pressure hydrocephalus and other kinds of secondary hydrocephalus (1–3).

An LPS is usually performed as a one-stage procedure, in which the patient's position does not need to be changed, or a two-stage procedure, in which the patient's position is changed. During the one-stage procedure, the shunt system is implanted while the patient is in a lateral position (2, 4), whereas during the two-stage procedure, the lumbar catheter is implanted first while the patient is in a lateral or prone position. The peritoneal catheter is implanted with the patient in the supine position (5, 6). As the patient's position must be changed, the operative sites are sterilized again, and the patient is redraped (6). These extra steps can increase the risk of surgical site infection and prolong the operative time. It is occasionally difficult to perform laparotomy using the one-stage procedure, particularly in obese patients. Laparoscopic-assisted laparotomy is now a popular method for this problem (7). However, neurosurgeons require help from general surgeons to perform laparoscopic-assisted laparotomy, and this may be difficult in some institutes. We designed a new continuous two-stage procedure for LPS, in which the patient's position is changed without re-sterilization or changing the surgical drapes. In this study, we analyzed the technical features and outcomes of this LPS procedure.

## Methods

We reviewed patients who underwent LPS using the continuous two-stage procedure from October 2019 to August 2021 in our institute. The patient's demographics and clinical features were collected. This study protocol was approved by the Ethics Committee of the First Affiliated Hospital, College of Medicine, Zhejiang University based on the ethical standards of the 1964 Declaration of Helsinki and its later amendments or comparable ethical standards.

Before the operation, all patients underwent cerebral and lumbar computed tomography/magnetic resonance imaging to evaluate hydrocephalus and the lumbar cistern. Furthermore, a lumbar puncture with the neck compression test was performed to confirm a fluent subarachnoid space. Cases of obstructive hydrocephalus and hydrocephalus with high intracranial pressure (ICP, > 200 mmH_2_O) were excluded from the patients who underwent LPS. All patients underwent LPS under general anesthesia utilizing a Strata adjustable pressure valve (Medtronic Inc., Minneapolis, MN, USA). The initial valve pressure was determined based on the ICP detected *via* a lumbar puncture before LPS and the patient's height and weight. Further adjustment was made according to the patient's clinical manifestations and neurological images. All patients underwent abdominal/lumbar computed tomography scanning to confirm the positions of the abdominal and lumbar catheters within 1 week after the operation.

In this study, we analyzed the LPS surgical duration using our method, the revision rate, and malfunctions in the shunt system within 90 days after the operation. Clinical and neuroimaging improvements were also analyzed.

### Continuous two-stage LPS

**Figure 1:**
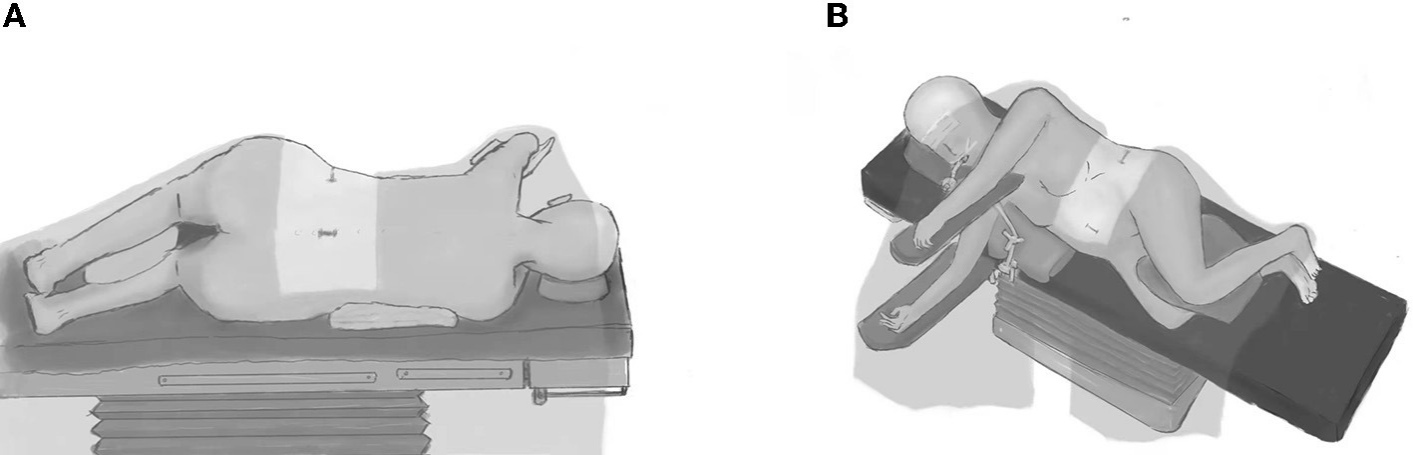
The lateral position before transition **(A)** posterior views; **(B)** anterior views. The patient is positioned in a right/left lateral position. Legs are curved for easy lumbar puncture, and the upper arm is fixed on the holder. Three incisions are marked, and then the surgical sites are sterilized and covered by surgical drapes.

The patient was set in a right/left lateral position (Figures 1A,B). A small cushion was placed under the lower arm, and another one was placed between the two curved legs. The three incisions (lumbar, anterior superior spine, and abdominal) were marked before sterilization. We do not usually use holders (lumbar and pubic symphysis) to immobilize the patient, but we fixed the upper arm in a holder (Figures 1A,B). After sterilization and covering the patient with surgical drapes, we used an adhesive surgical film to cover the surgical sites and fix the drapes.

We implanted the lumbar catheter and shunt valve (placed on the anterior superior spine site) during the operation and connected the lumbar catheter, shunt valve, and abdominal catheter. The abdominal catheter was drawn from the abdominal incision (paraumbilical skin incision). We closed the incisions at the lumbar and anterior superior spine and covered them with surgical drapes.

**Figure 2:**
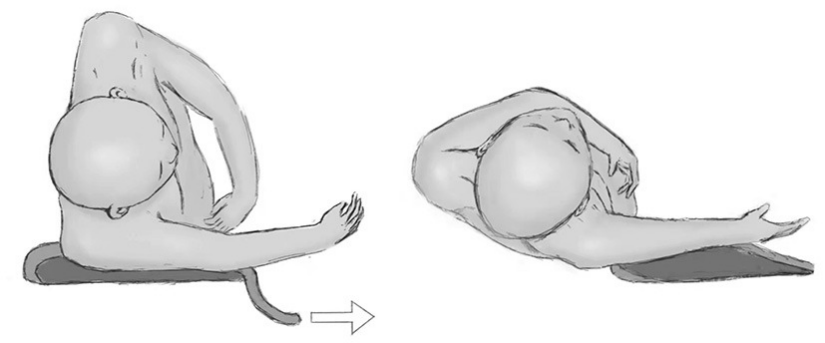
The transition of the patient's position. The nurse first straightens the patient's legs and releases the fixed upper arm. Then, the nurse pulls the cushion under the lower arm, and meanwhile, the surgeon pushes the patient's back to transform into a supine position.

**Figure 3:**
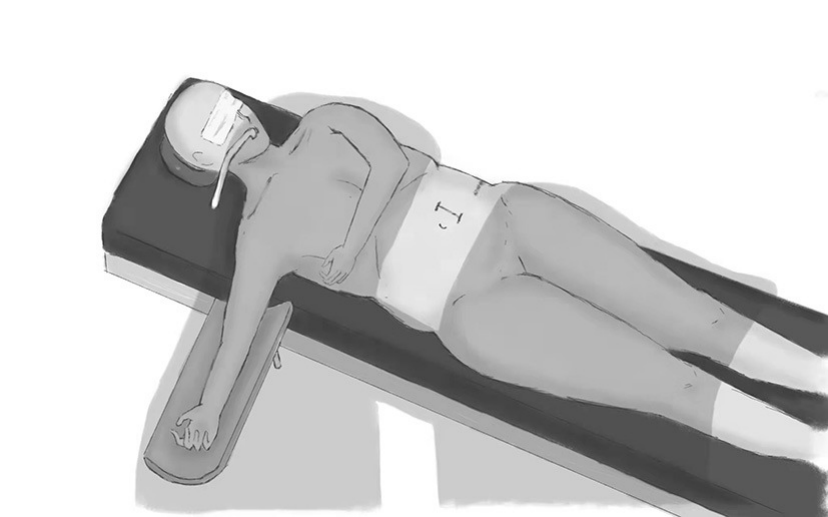
Supine position after transformation. After the transformation, the patient is close to a supine position for further laparotomy. We can tilt the surgical bed if the patient's position is not satisfactory.

The nurse straightened the patient's legs and released the upper arm fixed in the holder. The nurse pulled the cushion from under the lower arm of the patient, and meanwhile, the neurosurgeon pushed the patient back to complete the change in the patient's position (Figure 2). The patient was close to the supine position (Figure 3) in which laparotomy can easily be performed. A video has been made for the introduction of this process (Supplementary Video 1). These procedures did not require removing the surgical drapes or re-sterilizing the surgical site, but the neurosurgeon who had pushed the patient changed their surgical gown and gloves.

## Results

A total of 46 patients (34 men and 12 women) underwent continuous two-stage LPS from October 2019 to June 2021. Among them, 33 developed normal pressure hydrocephalus (NPH) after head trauma, 9 developed NPH after intracranial hemorrhage, and 2 were diagnosed with idiopathic normal pressure hydrocephalus (iNPH).

All of the LPS procedures were completed by one surgical team, and two or three neurosurgeons participated in each operation. The mean operative duration was 70.6 ± 12.7 min. The 180-day revision rate for these patients was 2.2% (1/46). A malfunction in the LPS system was detected in one patient within 180 days after the operation. The patient developed an intraventricular hemorrhage after LPS. Although the hemorrhage did not need surgical management, the shunt system was obstructed by bloody cerebrospinal fluid (CSF). He underwent a revision operation to change the valve. No patient developed a shunt infection in this series.

Of the patients, 76.1% (35/46) experienced clinical improvement after LPS during the 180-day follow-up, and 70.0% (32/46) experienced neuroimaging improvement.

## Discussion

Neurosurgeons do not need to open the cranium or puncture a lateral ventricle when performing LPS, unlike VPS, which means a lower risk of intracranial hemorrhage. Subdural effusions and hematomas are frequent postoperative complications secondary to LPS; however, these complications are as likely to occur after LPS and other CSF shunt procedures with the development of the shunt system and surgical technique (1). In a study comparing LPS and VPS among patients with iNPH, the two procedures had comparable rates of shunt failure and complications (8). LPS is probably a safer choice than VPS for cases of hydrocephalus secondary to severe head trauma or for patients who have a history of repeated idiopathic intracerebral hemorrhage. In our series, most of the patients underwent LPS because of secondary hydrocephalus. A number of these patients were still unconscious when undergoing LPS, and the tracheotomy tube was maintained. The LPS surgical site is distant from the tracheotomy incision, which is helpful to reduce the risk of infection. Furthermore, the outcomes of LPS are similar to those of other treatments, including VPS and ventriculoatrial shunt (VAS), among patients with iNPH (1, 9), and secondary hydrocephalus (2, 10). As a result, we preferred LPS at our institute for managing secondary hydrocephalus after the evaluation process.

Lumboperitoneal shunt can be performed *via* a one-stage or two-stage procedure. In the one-stage procedure, LPS is completed in the lateral position, in which the patient's position is not changed during the operation. However, it is difficult to perform a laparotomy with the patient in the lateral position, particularly in obese patients. Laparoscopic-assisted laparotomy is an easy strategy to solve this problem (7, 11–13). However, laparoscopic-assisted laparotomy usually requires participation by an abdominal surgeon, and this may be a problem in some institutes. Besides, although increased abdominal pressure during laparoscopic surgery usually does not affect ICP or the CSF shunt system in the presence of a working unidirectional valve, it should be a concern when the hydrocephalus is highly shunt-dependent (14). Goto et al. described lateral abdominal laparotomy, excluding laparoscopic-assisted laparotomy (6). They made an incision between the 10^th^ costal inferior edge and the anterior superior iliac spine and divided the muscles using a blunt dissector to insert the distal catheter. With this method, the mean operative time was 38.82 ± 13.87 min; and only one patient developed a secondary complication post-operation. Kawahara et al. designed blunt scalp hooks with a rubber ring to retract the operative field during the LPS procedure, with the patient in a lateral position (4). Over a 3-year period, the authors reported 12 out of 125 patients who developed complications.

The patient's position must be changed for the two-stage procedure to allow access to the lumbar theca in the lateral or prone position and the peritoneal end in the supine position. After moving the patient, the operative sites should be sterilized again and covered with new surgical drapes (6). These extra steps may increase the risk of surgical site infection and prolong the operative time. In this study, we designed a two-stage LPS procedure in which the surgical drapes were not changed. The mean operative duration was 70.6 ± 12.7 min in this series, and only one patient required a revision operation within 180 days post-LPS. No patient in this series developed a shunt infection, although the follow-up time was limited. Similar to our method, Shimizu et al. introduced a transportation board to facilitate changing the patient's position and keep the surgical drapes in place (15). Compared with a one-stage procedure, the two-stage procedure commonly needs longer operative time; however, this method appears to be simpler to carry out and has a low incidence of surgical complications. In this study, only one patient underwent revision of the shunt among 46 patients (2.2%). In addition, Yang et al. also reported a low rate of revision (1.0%) among 96 patients who underwent a two-stage LPS (5).

This continuous two-stage LPS procedure is easily performed. The nurse must straighten the patient's legs before changing their position. Then, the patient is close to the supine position. Moreover, the neurosurgeon who pushes the patient back changes their surgical clothes to guarantee a sterile operation.

In this series, LPS performed using a continuous two-stage procedure had low rates of shunt malfunction and infection. However, the small number of patients and retrospective design are limitations of this study.

## Conclusion

We described a continuous two-stage LPS procedure, in which the operative site did not need to be sterilized again, and the surgical drapes did not need to be changed after changing the patient's position. This method simplified the two-stage LPS procedure and had low rates of shunt malfunction and infection.

## Data availability statement

The raw data supporting the conclusions of this article will be made available by the authors, without undue reservation.

## Ethics statement

The studies involving human participants were reviewed and approved by the Ethics Committee of the First Affiliated Hospital, College of Medicine, Zhejiang University. Written informed consent for participation was not required for this study in accordance with the national legislation and the institutional requirements. Written informed consent was obtained from the individuals for the publication of any potentially identifiable images or data included in this article.

## Author contributions

ZL, HW, HZ, and JY: data collection and writing paper. XY: study design. LW: study design, data collection, and writing paper. All authors contributed to the article and approved the submitted version.

## Funding

This work was supported by Medical Health Science and Technology Project of Zhejiang Provincial Health Commission (2021KY1169), the Basic Public Welfare Research Program of Zhejiang Province (LGF21H090010), and the National Natural Science Foundation of China (NFSC No. 81971159).

## Conflict of interest

The authors declare that the research was conducted in the absence of any commercial or financial relationships that could be construed as a potential conflict of interest.

## Publisher's note

All claims expressed in this article are solely those of the authors and do not necessarily represent those of their affiliated organizations, or those of the publisher, the editors and the reviewers. Any product that may be evaluated in this article, or claim that may be made by its manufacturer, is not guaranteed or endorsed by the publisher.
