# Corrigendum: Lumboperitoneal shunt surgery *via* continuous two-stage procedure: Technique notes and outcomes

**DOI:** 10.3389/fneur.2023.1156059

**Published:** 2023-02-27

**Authors:** Zhao Li, Hao Wang, Han Zhang, Jiqi Yang, Xiaofeng Yang, Liang Wen

**Affiliations:** ^1^Shengzhou Hospital of Traditional Chinese Medicine, Shengzhou, China; ^2^First Affiliated Hospital, School of Medicine, Zhejiang University, Hangzhou, Zhejiang, China; ^3^Shengzhou People's Hospital, First Affiliated Hospital, School of Medicine, Zhejiang University, Shengzhou, China

**Keywords:** hydrocephalus, lumboperitoneal shunt, rehabilitation, shunt implantation, cerebrospinal fluid

In the published article, there was an error in the **Funding** statement. The incorrect funding number was used for the Medical Health Science and Technology Project of Zhejiang Provincial Health Commission. The correct Funding statement appears below.

